# On the genus *Polypedilum*, subgenus Collartomyia, with description of P. (Col.) baishanzuensis sp. nov. from Baishanzu Nature Reserve, China (Diptera, Chironomidae)

**DOI:** 10.3897/zookeys.1065.69870

**Published:** 2021-10-22

**Authors:** Chao Song, Binqing Zhu, Wei Liu, Xin Qi

**Affiliations:** 1 College of Life Sciences, Taizhou University, Taizhou, Zhejiang 318000, China Taizhou University Taizhou China; 2 Nanjing Institute of Environmental Sciences, Ministry of Ecology and Environment, Nanjing 210042, Jiangsu, China Nanjing Institute of Environmental Sciences Nanjing China; 3 Lishui Baiyun Ecological Forest Farm, Lishui 323000, Zhejiang, China Lishui Baiyun Ecological Forest Farm Zhejiang China

**Keywords:** Chironominae, Collartomyia, DNA barcode, key, morphology, new species, *Polypedilum*, taxonomy

## Abstract

A new species of the genus *Polypedilum* Kieffer, 1912 is described from Baishanzu Nature Reserve, China, based on molecular and morphological data. Molecular phylogenetic analysis based on standard barcode sequences confirmed a new clade of Polypedilum (Collartomyia) species. The new species is easily distinguished from its congeners by a combination of the following morphological characters: membrane of wing with a large spot occupying 70% of the proximal area; tergite without dark brown band pigmentation; tarsi I–V dark brown; superior volsella with three outer lateral setae and six long setae along inner base; inferior volsella with setose tubercules. An updated key to adult males of the subgenus Collartomyia is also provided.

## Introduction

*Polypedilum* Kieffer, 1912 is the largest chironomid genus, with more than 520 known species worldwide. Its subgeneric divisions and phylogeny have always been disputable and intractable (Sæther et al. 2010; Cranston et al. 2016; Yamamoto et al. 2016; Pinho and Silva 2020; Tang et al. 2021). Only two subgenera, *Collartomyia* Goetghebuer, 1936 and *Tripodura* Townes, 1945, form certain monophyletic groups. The subgenus Collartomyia was recently recognized by Tang et al. (2021) for the species having wing with a brownish band or dark spots, a well-developed gonocite bulb, split setae usually present on inner margin of gonostylus, including the previous subgenus Cerobregma Sæther & Sundal, 1998 and the monotypic genus Yaethauma Yamamoto, Yamamoto & Tang, 2018. The subgenus now includes 21 valid species recorded in the Afrotropical, Holarctic, and Oriental regions (Sæther and Sundal 1999; Kobayashi et al. 2003; Zhang and Wang 2005; Zhang et al. 2006; Moubayed-Breil 2007; Tang and Niitsuma 2017; Yamamoto et al. 2018; Lin et al. 2019; Qi et al. 2020; Liu et al. 2021).

DNA barcoding provides an effective and quick tool for species identification and delimitation, and has been proven successful in many different kinds of animals (Herbert et al. 2003). Chironomid researchers around the world have uploaded 3,310 species including 599,223 sequences in the Barcode of Life Database (BOLD) before 16 June, 2021. Barcode sequences are becoming a necessary character for chironomid species identification and new species descriptions (Song et al. 2016, 2018; Lin et al. 2018, 2020; Makarchenko et al. 2020; Qi et al. 2020).

Baishanzu National Nature Reserve is located in the south Zhejiang and north Fujian provinces of China; this region is well known for its high level of biodiversity and hot spots in Asia. It belongs to the tropical to warm temperate transitional zone. During field surveys in Baishanzu Nature Reserve, an unknown species of the genus *Polypedilum* were collected. Molecular data and morphological comparisons supported it as an undescribed taxon that we describe herein as a new species.

## Material and methods

The examined material was collected by light trap and then preserved in 75% ethanol at 4 °C in a refrigerator before final slide mounting. Tissues for total genomic DNA extraction were removed from the thorax and head of the adults. The extraction procedure followed the Qiagen DNeasy Blood and Tissue kit guide except for the use of an elusion buffer quantity of 120 µl. After extraction, the exoskeletons were cleared and mounted on corresponding slides following the procedure described by Sæther (1969). Morphological terminology follows that of Sæther (1980). The photograph of the dorsal habitus was obtained with a DV500 5MP Digital Camera attached to a stereo microscope (Chongqing Optec SZ680). The photograph of the body parts was obtained using a Leica DMLS compound microscope. Photograph post-processing was done in Adobe photoshop and Illustrator (Adobe Inc., California, USA).

Abbreviations used are as follows:

**AR** antennal ratio;

**BR** bristle ratio;

**BV** beinverhältnisse;

**Cu** cubitus;

**Dc** dorsocentrals;

**Fe** femur;

**HR** hypopygium ratio;

**HV** hypopygium value;

**IV** inner verticals;

**LR** leg ratio;

**M** media;

**MCu** crossvein between media and cubitus;

**OV** outer verticals;

**Pa** prealars;

**Po** post orbitals;

**R** radius;

**RM** crossvein between radius and media;

**Ta** tarsomere;

**Ti** tibia;

**VR** venarum ratio.

The standard barcode region of COI-5P was amplified using the universal primers LCO1490 and HCO2198 (Folmer et al. 1994). PCR amplifications were carried out in a 25 μl volume including 12.5 μl 2 × Es Taq MasterMix (CoWin Biotech Co., Beijing, China), 0.625 μl of each primer, 2 μl of template DNA and 9.25 μl deionized H_2_O following Song et al. (2018). PCR products were electrophoresed in 1.0% agarose gel, purified, and sequenced in both directions using an ABI 3730XL capillary sequencer (Beijing Genomics Institute Co., Ltd., Hangzhou, China). Raw sequences were assembled into contigs and edited in BioEdit 7.2.5 (Hall 1999). The pairwise distances were calculated using the Kimura 2-Parameter (K2P) substitution model in MEGA 7(Kumar et al. 2016). The neighbor joining tree was constructed using the K2P substitution model, 1,000 bootstrap replicates and the “complete deletion” option for missing data. Sequences, trace-files, and metadata of the new species were uploaded to the Barcode of Life Data Systems (BOLD) (Ratnasingham and Hebert 2013).

## Results

### Barcode analysis

**Figure 1.**
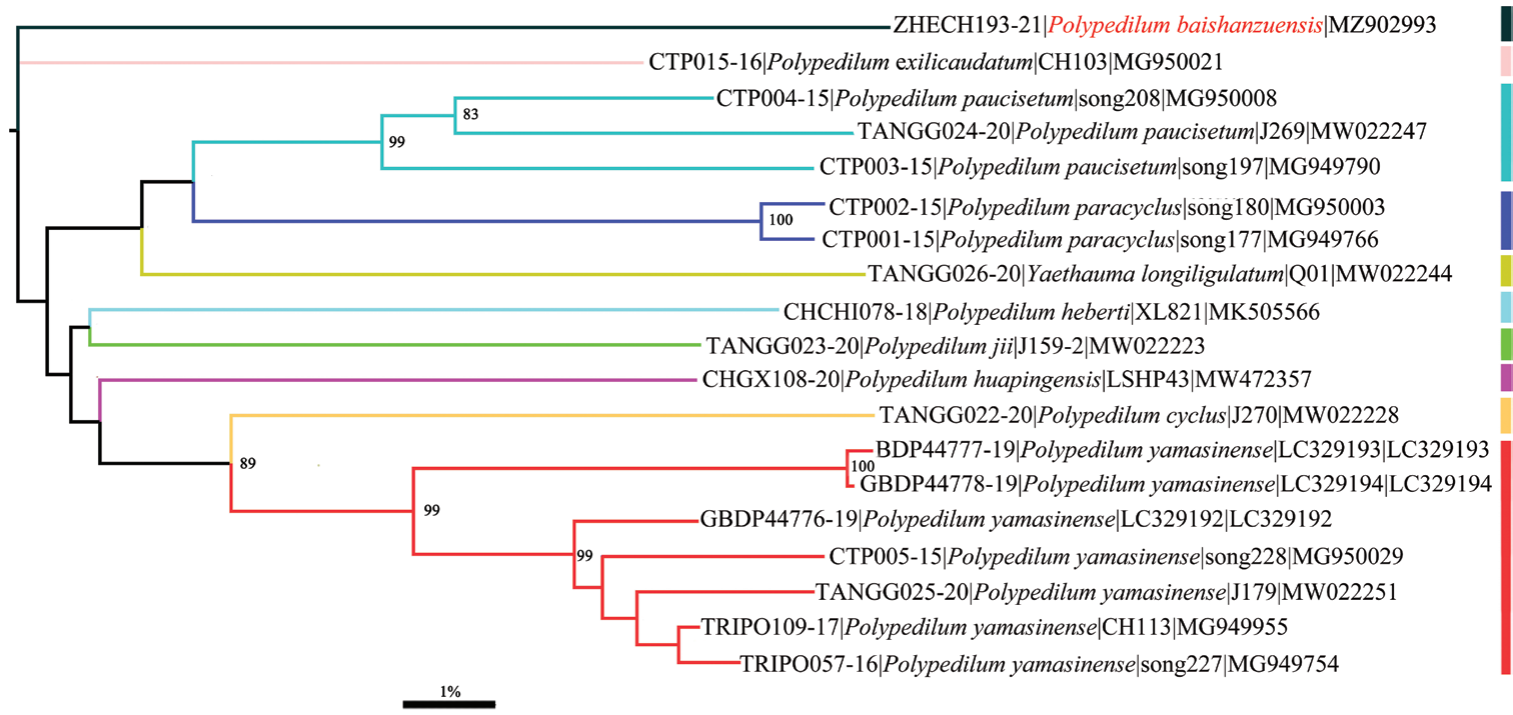
Neighbor joining tree of 10 species of Polypedilum (Collartomyia) COI barcodes based on K2P model. Numbers on branches represent bootstrap support (> 75%) based on 1,000 replicates, scale represents K2P genetic distance.

The species was primarily blasted in GenBank and molecularly confirmed as a species of *Polypedilum*. Morphological characters support it belonging to the subgenus Collartomyia. All ten species with public COI sequences of P. (Collartomyia) species were used to construct the neighbor-joining tree based on COI barcode sequences and a distinct genetic branch suggests that our specimen belongs to a species new to science (Fig. 1). The minimum interspecific genetic distance within the subgenus Collartomyia is up to 14.8% divergence in partial COI sequences (Table 1), larger than the 5–8% threshold suggested by Song et al. (2016, 2018). The genetic divergence to the morphologically similar species Polypedilum (Collartomyia) heberti Lin & Wang, 2018 and Polypedilum (Collartomyia) huapingensis Liu & Lin, 2021 are up to 15.9% and 15.1% divergent, respectively.

**Table 1.** Kimura 2-parameter pairwise genetic distances based on COI barcodes of Polypedilum (Collartomyia).

Species	Pairwise genetic distances																	
*P.baishanzuensis*|BSZ60																		
*P.cyclus*|MW022228	17.5																	
*P.exilicaudatum*|MG950021	14.8	15.6																
*P.heberti*|MK505566	15.8	15.2	13.6															
*P.huapingensis*|MW472357	14.8	13.0	13.0	13.0														
*P.jii*|MW022223	15.5	13.8	13.0	12.8	12.6													
*P.longiligulatum*|MW022244	16.9	16.6	14.7	16.0	14.4	14.0												
*P.paracyclus*|MG949766	17.3	16.0	13.4	14.2	13.8	14.2	14.4											
*P.paracyclus*|MG950003	17.1	15.1	14.4	14.4	13.8	14.4	14.6	1.2										
*P.paucisetum*|MW022247	17.7	15.6	13.2	15.6	14.7	14.2	13.4	13.2	13.8									
*P.paucisetum*|MG949790	17.3	13.8	13.2	15.2	15.3	14.8	13.2	12.0	12.2	9.0								
*P.paucisetum*|MG950008	14.8	14.0	11.6	14.2	14.0	12.4	12.2	10.7	11.1	6.5	7.4							
*P.yamasinense*|MG949955	16.0	11.5	13.4	12.8	12.2	12.2	13.4	13.8	14.0	14.5	14.4	14.1						
*P.yamasinense*|MW022251	16.2	13.0	13.0	14.2	13.0	13.4	14.6	15.8	15.8	16.2	16.0	15.1	2.7					
*P.yamasinense*|LC329192	15.4	11.6	13.2	12.4	11.8	12.8	13.4	13.6	13.8	15.4	13.8	13.8	2.0	3.5				
*P.yamasinense*|LC329193	18.5	12.0	13.8	15.0	14.5	14.4	15.6	14.2	13.8	16.8	16.2	15.8	6.7	8.1	7.4			
*P.yamasinense*|LC329194|	18.3	11.8	13.6	14.8	14.3	14.2	15.4	14.0	14.0	16.6	16.0	15.5	6.5	7.9	7.2	0.3		
*P.yamasinense*|MG949754	16.2	12.4	13.6	13.0	12.4	12.8	14.2	14.4	14.6	15.6	15.0	14.8	0.8	2.5	2.2	7.6	7.4	
*P.yamasinense*|MG950029	16.2	12.6	13.9	14.2	13.2	13.2	15.4	15.6	15.4	14.9	15.4	14.3	3	4.7	4.0	8.9	8.7	2.8

### Morphological description

#### Polypedilum (Collartomyia) baishanzuensis

Taxon classificationAnimaliaDipteraChironomidae

Song & Qi sp. nov.

B0435B89-388C-5646-92EF-8058A1F1F708

http://zoobank.org/F43550CB-6444-4F86-9F41-09D2121DD6C1

Figs 2 , 3 , 4

##### Type material.

***Holotype*** (BOLD & TZU sample ID: ZJCH193; Field ID: BSZ60) 1 ♂, China, Zhejiang Province, Lishui City, Qingyuan county, Baishanzu National Nature Reserve, 27.76°N, 119.31°E, 11.VIII.2020, Qi X., light trap.

The holotype is deposited in the collection of the College of Life Sciences, Taizhou University, Taizhou, China (TZU).

##### Diagnostic characters.

The male adult can be distinguished from other P. (Collartomyia) species by the following combination of characters: most of the body yellowish; wing with distinct spots on 70% of the proximal part; tarsomeres dark brown; tergite without dark brown band pigmentation; superior volsella with six inner basal setae and three outer lateral setae; dorsal side of inferior volsella with three distinct setiferous tubercles.

##### Etymology.

The specific name refers to the Baishanzu National Nature Reserve, where the holotype was collected.

Adult male (n = 1). Total length 4.40 mm; wing length 2.75 mm; total length / wing length 1.60; wing length / length of profemur 2.11.

**Figure 2.**
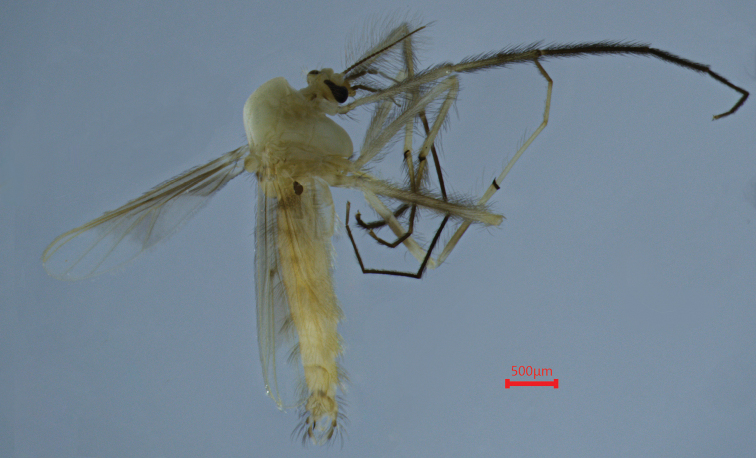
Holotype male of Polypedilum (Collartomyia) baishanzuensis Song & Qi, sp. nov.

***Coloration*** (Fig. 2). Head, thorax and abdomen yellowish; palpomeres dark brown to blackish; femur, tibia and tarsomeres ta1–ta5 of P1 blackish; tarsomeres ta1-ta5 of P2 dark brown; tarsomeres ta3–ta5 of P3 dark brown; gonocoxite and proximal half of gonostylus dark brown.

**Figure 3.**
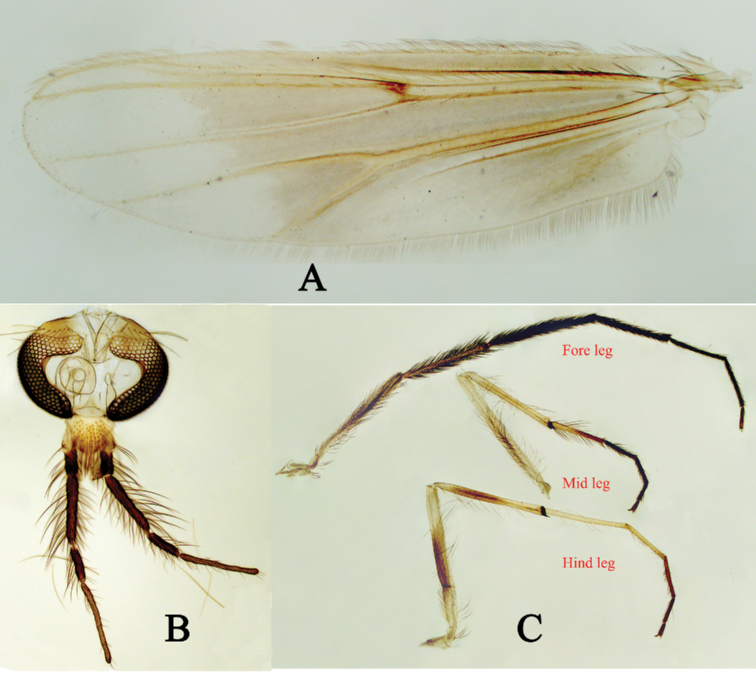
Holotype male of Polypedilum (Collartomyia) baishanzuensis Song & Qi, sp. nov. **A** wing **B** head **C** legs.

***Head*** (Fig. 3B). Frontal tubercle absent. Antenna with 13 flagellomeres, ultimate flagellomere 480 µm long; AR 0.77. Temporal setae 16, including 8 inner verticals and 8 outer verticals; Clypeus with 57 setae; Palpomere lengths (in μm): 70, 95, 277, 145, 330. Length of 5^th^ palpomere / 3^rd^ palpomere 1.19.

***Thorax***. Dorsocentrals 50; acrostichals 8; prealars 16; scutellum with 39 setae.

***Wing*** (Fig. 3A). VR 1.14; Brachiolum without setae; R with 32 setae; R_1_ with 44 setae; R_4+5_ with 69 setae; Squama with 33 setae. Anal lobe moderately developed.

***Legs*** (Fig. 3C). Terminal scale of fore tibia pointed, 37 μm long. Spur of mid tibia 55 μm long including 32 tooth comb, unspurred comb with 33 teeth. Spur of hind tibia 65 μm long including 26 teeth, unspurred comb with 32 teeth. Lengths (in µm) and proportions of legs as in Table 2.

**Table 2.** Lengths (in µm) and proportions of legs of holotype male of Polypedilum (Collartomyia) baishanzuensis sp. nov. (n = 1).

	Fe	Ti	Ta_1_	Ta_2_	Ta_3_	Ta_4_	Ta_5_	LR	BV	SV	BR
P1	1300	1010	1250	850	660	550	265	1.24	0.65	1.85	3.03
P2	1450	1150	640	395	305	200	150	0.56	0.32	4.06	3.90
P3	1600	1260	960	550	430	280	160	0.77	0.37	2.98	2.90

**Figure 4.**
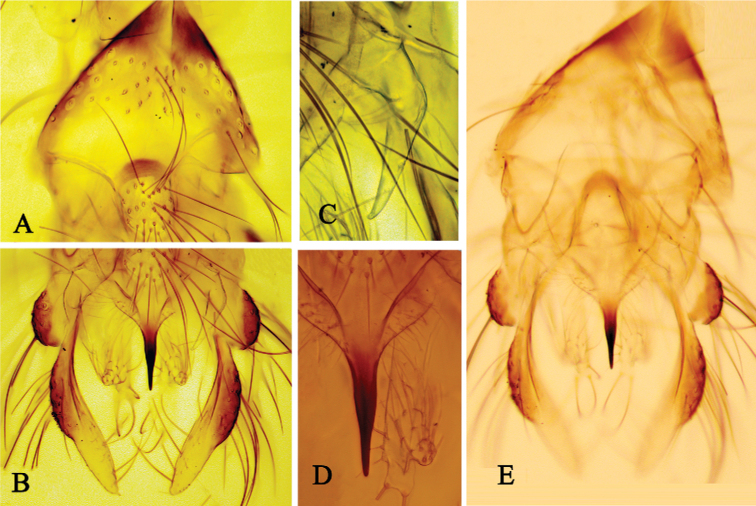
Holotype male of Polypedilum (Collartomyia) baishanzuensis Song & Qi, sp. nov. **A** tergite VIII **B** hypopygium, dorsal view **C** superior volsella **D** inferior volsella **E** hypopygium, ventral view.

**Figure 5.**
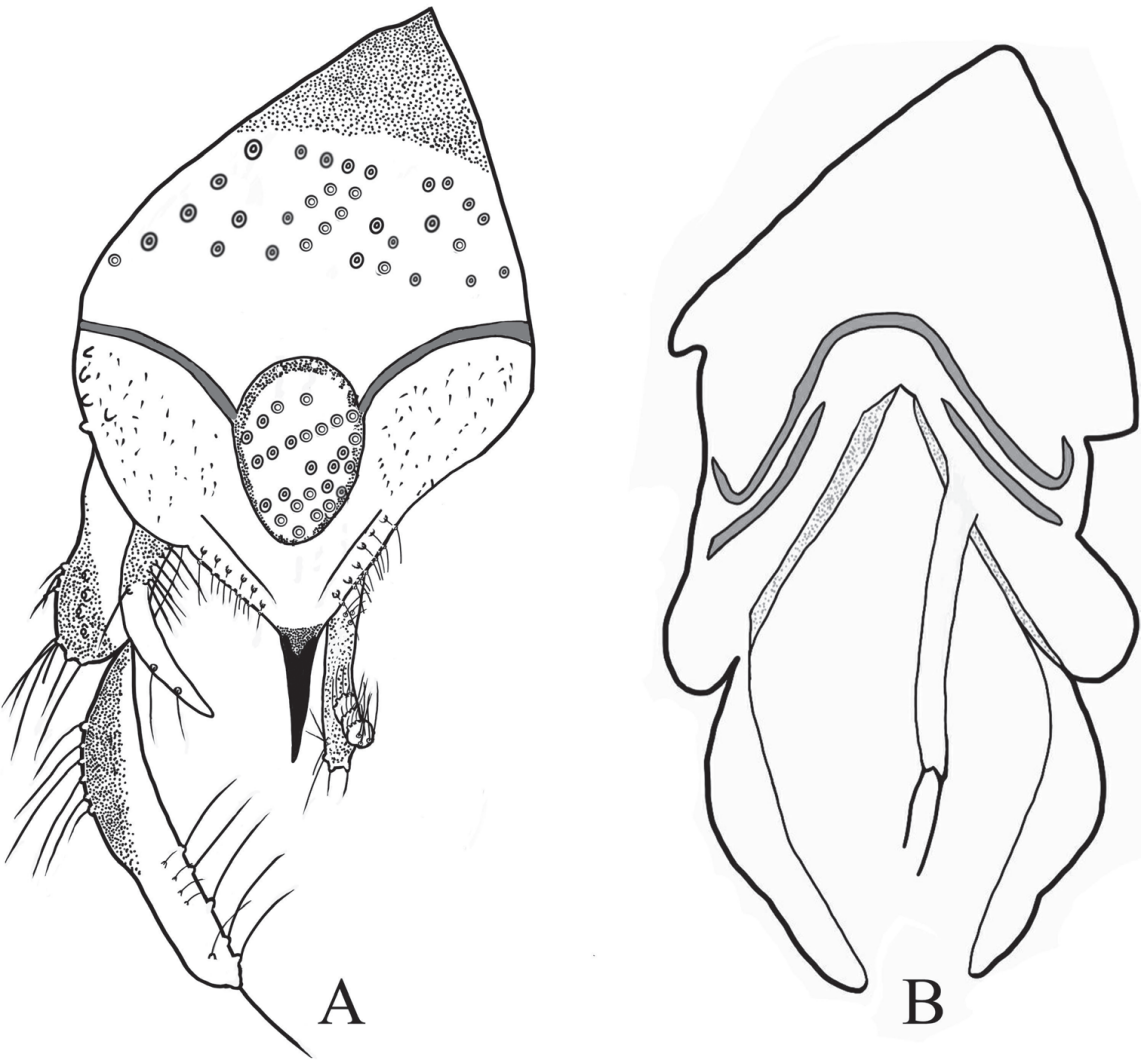
Holotype male of Polypedilum (Collartomyia) baishanzuensis Song & Qi, sp. nov. **A** hypopygium, dorsal view **B** hypopygium, ventral view.

***Hypopygium*** (Figs 4–5). Basal part of abdominal segment VIII distinctly triangular and markedly pointed at base (Fig. 4A, 4E). Anal tergite with 27 median setae, laterosternite with 5 setae; Anal tergite bands strongly developed and sclerotized, forming a circle completely surrounding median setae. Anal point (Fig. 4B) 125 μm long and 27.5 μm wide at base, 5 μm at apex; transverse sternapodeme 112 μm long, phallapodeme 175 μm long. Basal part of superior volsella (50 μm long and 50 μm wide) covered with microtrichia and with 6 long setae along inner base and one long seta on outer side; projecting part of superior volsella 105 μm long, with 2 long setae along the distal outer part (Fig. 4C). Inferior volsella (Fig. 4D) 217 μm long, with 3 tubercle-like projections with strong macrosetae. Gonostylus 262 μm long with macrosetae along distal inner margin. HR 1.0. HV 1.69.

Immatures and female unknown.

##### Ecology.

**Figure 6.**
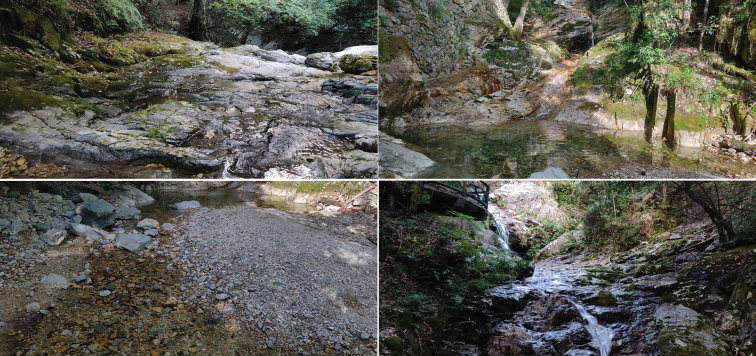
Type locality of Polypedilum (Collartomyia) baishanzuensis Song & Qi sp. nov.

Material composed of male adults was light-trapped from stones and boulders in a flowing mountain stream (Fig. 6), located at an altitude of 1,450 m.

##### Distribution.

Only known from the type locality in Zhejiang province, China.

## Discussion

The morphological characters of the well-developed gonocoxite bulb of the new species clearly fit the subgeneric definition by Tang et al. (2021) and Sæther and Sundal (1998). The new species shows close morphological similarity with other P. (Collartomyia) species on the basis of the spotted wings, including those of P. (C.) heberti Lin & Wang, 2018 and P. (C.) huapingensis Liu & Lin, 2021, but can be clearly distinguished by tergite IX without a dark brown band or spots, tarsomeres of P1 dark brown, inferior volsella present, with three dorsal setiferous tubercles. Other differences are listed in Table 3.

**Table 3.** Main differences between P. (C.) heberti, P. (C.) huapingensis, and P. (C.) baishanzuensis sp. nov.

Species	AR	LR1	Ta. of P1	Anal point	SVo	Ivo, dorsal side
*P.baishanzuensis*	0.77	1.24	dark brown	tapering	6 inner setae 3 outer setae	setose tubercles, present
*P.heberti*	0.51	1.02	yellow	tapering	5 inner setae outer setae	tubercles, absent
*P.huapingensis*	0.44	2.17	yellow	broadening	2 inner setae 1 outer seta	tubercles, absent

### Key to known adult males of the Polypedilum (Collartomyia) modified from Lin et al. (2019) and Sæther and Sundal (1999)

**Table:** 

1	Antepronotal lobes reduced, with elongate scutal projection	**2**
–	Antepronotal lobes narrowed dorsally and medially narrowly separated	**4**
2	Maxillary palp reduced	***P.hirsutum* (Goetghebuer)**
–	Maxillary palp five-segmented	**3**
3	Antepronotal lobe distinctly narrowed dorsally	***P.longiligulatum* Yamamoto, Yamamoto & Tang**
–	Antepronotal lobe reduced, with anteriorly elongate scutal projection	***P.discaudatum* Amakye**
4	Wing with dark spots	**5**
–	Wing without spots	**11**
5	Palpomeres reduced, palpomeres 4 and 5 combined about as long as palpomere 3; Sudan	***P.brevipalpe* Sæther & Sundal**
–	Palpomeres five-segmented, fifth palpomere about twice as long as third palpomere	**6**
6	Antepronotum setose	**7**
–	Antepronotum bare	**8**
7	Superior volsella with two outer setae; France, Italy	***P.lotensis* Moubayed-Breil**
–	Superior volsella without outer setae; Ghana, Tanzania	***P.volselligum* Sæther & Sundal**
8	Wing with obvious spots; setae along inner margin of gonostylus strongly split	***P.ramiferum* Kieffer**
–	Wing with a large black spot on entire basal area; setae along inner margin of gonostylus not split	**9**
9	Anal point strong, mid-part contracted in a large inflated globe apically, with candle-like spine	***P.huapingensis* Liu & Lin**
–	Anal point strong and tapering	**10**
10	Tergites II–VI brown with dark brown bands at middle	***P.heberti* Lin & Wang**
–	Tergites II–VI pale brown without brown bands at middle	***P.baishanzuensis* Song & Qi sp. nov.**
11	Antepronotum setose	**12**
–	Antepronotum bare	**15**
12	Anal point broad, tapering towards apex; Canada and USA	***P.ontario* (Wally)**
–	Anal point narrow, parallel-sided	**13**
13	Apical process of superior volsella without strong outer seta in apical half	***P.okigrandis* Sasa**
–	Apical process of superior volsella with strong outer seta in apical half	14
14	Fore tibial scale pointed; tergite IX with strongly sclerotized circle; China	***P.cyclus* Zhang & Wang**
–	Fore tibial scale rounded; tergite IX without strongly sclerotized circle; China and Japan	***P.yamasinense* (Tokunaga)**
15	Scutum with a weak tubercle	**16**
–	Scutum without a tubercle	**17**
16	Superior volsella with one long outer seta; R_2+3_ distinct; China	***P.jii* Zhang & Wang**
–	Superior volsella without outer setae; R_2+3_ evanescent; China	***P.exilicaudatum* Sæther & Sundal**
17	Anal point broad, not parallel-sided; legs ringed with white	**18**
–	Anal point narrow, parallel-sided; legs not ringed	**19**
18	Anal point broad with strong median swelling and apical additional point; Ghana	***P.bulbocaudatum* Sæther & Sundal**
–	Anal point awl-shaped, without an additional apical point; Ghana	***P.subulatum* Sæther & Sundal**
19	Legs with dark patterns	***P.paracyclus* Qi & Song**
–	Legs without dark patterns	**20**
20	AR 0.54–0.91; tergite IX with two setae; superior volsella short and broad; China	***P.paucisetum* Zhang**
–	AR 1.15; tergite IX with more than 40 setae; superior volsella curved and tapered; France	***P. sætheri* Moubayed-Breil**

## Supplementary Material

XML Treatment for Polypedilum (Collartomyia) baishanzuensis
